# Cerebral Vasospasm After Burr Hole Evacuation of Chronic Subdural Hematoma

**DOI:** 10.7759/cureus.55140

**Published:** 2024-02-28

**Authors:** Masahiro Morishita, Takaaki Yamazaki, Makoto Senoo, Mikio Nishiya

**Affiliations:** 1 Department of Neurosurgery, Hakodate Neurosurgical Hospital, Hokkaido, JPN

**Keywords:** traumatic brain injury (tbi), subarachnoid hemorrhage, magnetic resonance angiography (mra), ischemic stroke, digital subtraction angiography (dsa), chronic subdural hematoma (csdh), cerebral vasospasm

## Abstract

Cerebral vasospasm is a frequent complication of subarachnoid hemorrhage. We report a case of chronic subdural hematoma complicated by cerebral vasospasm after burr hole evacuation. A 74-year-old woman underwent burr hole evacuation of a chronic subdural hematoma. She developed left hemiparesis and disturbance of consciousness on postoperative day 3. Magnetic resonance imaging showed a right parietal infarct and decreased cerebral blood flow signal in the right middle cerebral artery territory. Digital subtraction angiography showed multiple segmental narrowings of the right middle cerebral artery. Her neurological symptoms recovered with conservative treatment. Follow-up angiography showed improvement in the arterial narrowing, which finally led to a diagnosis of cerebral vasospasm. Cerebral vasospasm can occur after burr hole evacuation of chronic subdural hematoma. Magnetic resonance angiography is useful for determining the cause of postoperative neurological worsening in chronic subdural hematoma patients.

## Introduction

Cerebral vasospasm is a frequent complication of subarachnoid hemorrhage (SAH) and a major cause of death and disability [1]. Subdural hematoma involves bleeding between the dura mater and arachnoid membrane, representing an anatomically distinct form of intracranial hemorrhage compared to SAH. Herein, we report a case of chronic subdural hematoma (CSDH) complicated by cerebral vasospasm after burr hole evacuation. Magnetic resonance angiography (MRA) was useful in identifying the cause of postoperative neurological worsening of CSDH patients and determining the treatment strategy.

## Case presentation

**Figure 1:**
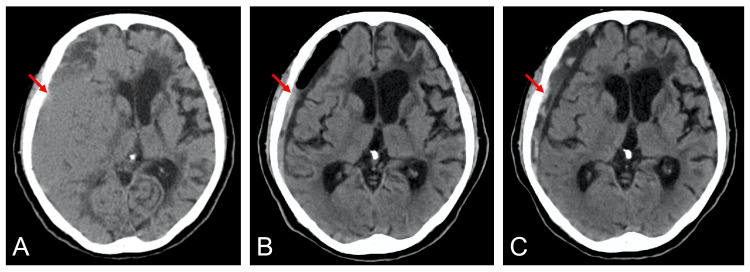
Head computed tomography Head computed tomography showing preoperative chronic subdural hematoma (A), and that on postoperative day 1 (B) and postoperative day 3 (C) (arrows)

A 74-year-old woman with a history of hypertension was transferred from another hospital with left hemiparesis and disturbance of consciousness. She had well-controlled blood pressure with a calcium channel blocker. Head computed tomography (CT) showed a 26-mm CSDH (Figure 1A). She underwent burr hole evacuation of the hematoma. The day after surgery, her consciousness and motor weakness recovered, and CT showed an obvious reduction in hematoma volume (Figure 1B). However, she developed left hemiparesis and disturbance of consciousness again on the morning of postoperative day 3. No significant increase in hematoma volume was shown on repeat CT (Figure 1C).

**Figure 2:**
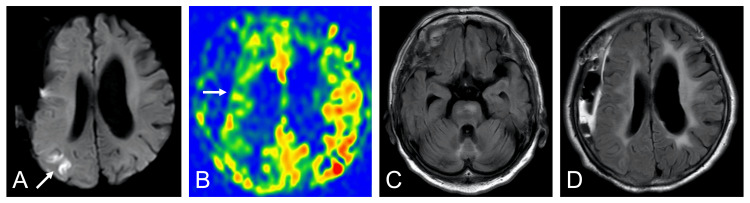
Magnetic resonance imaging Magnetic resonance imaging on postoperative day 4 showing a right parietal infarct on diffusion-weighted imaging (A) (arrow), decreased cerebral blood flow signal in the right middle cerebral artery territory on arterial spin labeling imaging (B) (arrow), and no subarachnoid hemorrhage on fluid-attenuated inversion recovery imaging (C, D)

**Figure 3:**
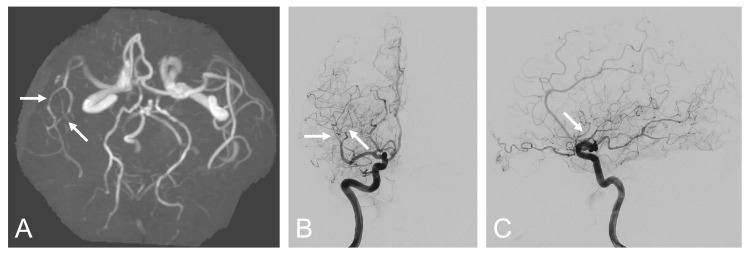
Magnetic resonance angiography and digital subtraction angiography Magnetic resonance angiography (A) and digital subtraction angiography (B, C) on postoperative day 4 showing segmental narrowings of the middle cerebral artery (arrows)

Magnetic resonance imaging showed a right parietal infarct, decreased cerebral blood flow signal in the right middle cerebral artery territory, and no evidence of SAH (Figure 2). MRA and digital subtraction angiography (DSA) showed multiple segmental narrowings of the right middle cerebral artery (Figure 3).

**Figure 4:**
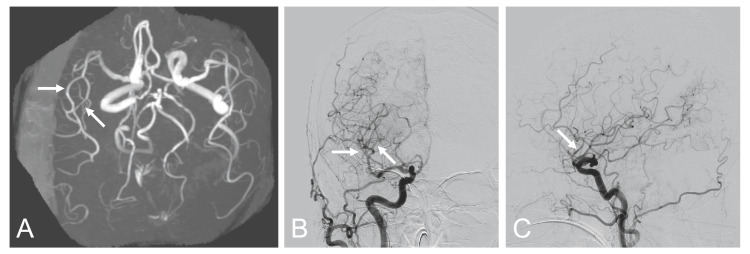
Follow-up magnetic resonance angiography and digital subtraction angiography Follow-up magnetic resonance angiography on postoperative day 8 (A) and digital subtraction angiography on postoperative day 17 (B, C) showing improvement in the arterial narrowings (arrows)

We reached a preliminary diagnosis of cerebral vasospasm and administered an intravenous crystalloid solution to maintain euvolemia and discontinued antihypertensive drug use for hemodynamic augmentation. On postoperative day 8, her neurological symptoms resolved with these conservative treatments. Follow-up MRA and DSA showed improvement in the arterial narrowing (Figure 4), which led to a final diagnosis of cerebral vasospasm.

## Discussion

The present case highlighted two important clinical issues. First, cerebral vasospasm can occur after burr hole evacuation of CSDH. Second, MRA is useful in identifying the cause of postoperative neurological worsening of CSDH patients and determining the treatment strategy.

Cerebral vasospasm can complicate aneurysmal or traumatic SAH and other conditions that cause bleeding into the subarachnoid space (e.g., ruptured vascular malformations, and hemorrhagic brain tumors) [2,3]. Cerebral vasospasm can be caused by spasmogenic substances from subarachnoid blood clots, which lead to endothelial damage and smooth muscle cell contraction [4]. However, cerebral vasospasm was also reported to occur in approximately 40% of patients with traumatic brain injury, while 24% of patients developed cerebral vasospasm without traumatic SAH [5]. Therefore, although SAH is an important risk factor for cerebral vasospasm in traumatic brain injury [6], other factors may also be involved in cerebral vasospasm.

Mechanical stretching and pulling of arteries, local inflammation, and spasmogenic substances released from the brain parenchyma injured by head trauma are thought to play an important role in the pathogenesis of posttraumatic cerebral vasospasm in the absence of SAH [7,8], although the effect of epidural or subdural hematoma, intracerebral hematoma or contusion on the development of cerebral vasospasm is unknown [6]. Our patient demonstrated severe cerebral vasospasm in the absence of SAH after burr hole evacuation of CSDH. Chronic compression of the brain parenchyma and arteries caused by CSDH, and sudden release of compression by hematoma evacuation, may stretch and injure arteries and parenchyma. This mechanical arterial stretching and release of spasmogenic substances from the injured parenchyma into the subarachnoid space may have caused cerebral vasospasm in our patient. To the best of our knowledge, only one similar CSDH case has been reported [9]. Furthermore, the mechanism of cerebral vasospasm in that case could be explained as posttraumatic cerebral vasospasm [5,6] because it was complicated by acute subdural hematoma. Thus, this is the first report of CSDH along with the development of cerebral vasospasm.

In the present study, cerebral angiography was useful in identifying the cause of postoperative neurological worsening in CSDH patients and in determining the treatment strategy. CSDH patients can experience postoperative neurological deterioration caused by recurrence, seizure, or stroke [10]. Although cerebral vasospasm can cause ischemic stroke [2] or mimic seizures [11]. Nevertheless, cerebral vasospasm is difficult to diagnose if cerebral angiography is not performed at the appropriate time because vasospasm is a transient phenomenon. Therefore, a missed diagnosis of cerebral vasospasm can unnecessarily lead to the use of permanent antithrombotic or antiepileptic drugs. In the present study, cerebral angiography was performed when postoperative neurological deterioration was observed, which allowed appropriate treatment. MRA is the first-choice imaging modality because of its capacity to detect cerebral vasospasm [12,13], noninvasiveness, and repeatability. The DSA and MRA findings were consistent in our case.

## Conclusions

This case illustrates cerebral vasospasm after burr hole evacuation of CSDH without SAH. MRA is useful in identifying cerebral vasospasm and affects treatment strategy in CSDH. MRA should be performed when postoperative neurological worsening is observed in CSDH. Some cerebral vasospasm associated with CSDH may not be recognized. Thus, hidden cerebral vasospasm may cause unexplained neurological worsening or ischemic stroke. Future studies are warranted to examine the prevalence and risk factors of cerebral vasospasm in CSDH.
